# Differential desulfurization of dibenzothiophene by newly identified MTCC strains: Influence of Operon Array

**DOI:** 10.1371/journal.pone.0192536

**Published:** 2018-03-08

**Authors:** Madhabi M. Bhanjadeo, Kalyani Rath, Dhirendra Gupta, Nilotpala Pradhan, Surendra K. Biswal, Barada K. Mishra, Umakanta Subudhi

**Affiliations:** 1 CSIR-Institute of Minerals and Materials Technology, Bhubaneswar, India; 2 Academy of Scientific and Innovative Research, New Delhi, India; 3 CSIR-Institute of Genomics and Integrative Biology, New Delhi, India; 4 Indian Institute of Technology Goa, Farmagudi, Ponda, India; Tallinn University of Technology, ESTONIA

## Abstract

Since the sulfur specific cleavage is vital for the organic sulfur removal from fossil fuel, we explored potential bacterial strains of MTCC (Microbial Type Culture Collection) to desulfurize the Dibenzothiophene (DBT) through C-S bond cleavage (4-S pathway). MTCC strains *Rhodococcus rhodochrous* (3552), *Arthrobacter sulfureus* (3332), *Gordonia rubropertincta* (289), and *Rhodococcus erythropolis* (3951) capable of growing in 0.5 mM DBT were examined for their desulfurization ability. The presence of *dsz* genes as well as the metabolites was screened by polymerase chain reaction (PCR) and HPLC, respectively. All these strains showed > 99% DBT desulfurization with 10 days of incubation in minimal salt medium. From the HPLC analysis it was further revealed that these MTCC strains show differences in the end metabolites and desulfurize DBT differently following a variation in the regular 4-S pathway. These findings are also well corroborating with their respective organization of *dsz*ABC operons and their relative abundance. The above MTCC strains are capable of desulfurizing DBT efficiently and hence can be explored for biodesulfurization of petrochemicals and coal with an eco-friendly and energy economical process.

## Introduction

Energy is essential to life, and its central source comes from fossil fuels. The elemental fossil fuels used today by most industrialized and developing countries are oil, coal, and natural gas [1, 2]. Among these fossil fuels, sulfur is the major contaminant contributing towards environmental pollution as well as health hazards in the form of acid rain and sulfur oxide emissions [3]. The current scenario of escalating inclination in global energy exploitation is due to mounting world population leading to more energy consumption. As a result, the highly efficient low sulfur fossil fuels are depleting. Therefore, we have to master the efficiency of the high sulfur fossil fuels for a sustainable energy source in coming future. At the same time retaining restricted sulfur emission as per the stringent rules for pollution control, the need of appropriate desulfurizing technologies for high sulfur-containing fossil fuels has become crucial. Among various sulfur bearing compounds the most predominant form of sulfur is dibenzothiophene (DBT) and its derivatives in the petroleum and coal [4, 5] which is refractory to the contemporary desulfurization techniques.

**Fig 1:**
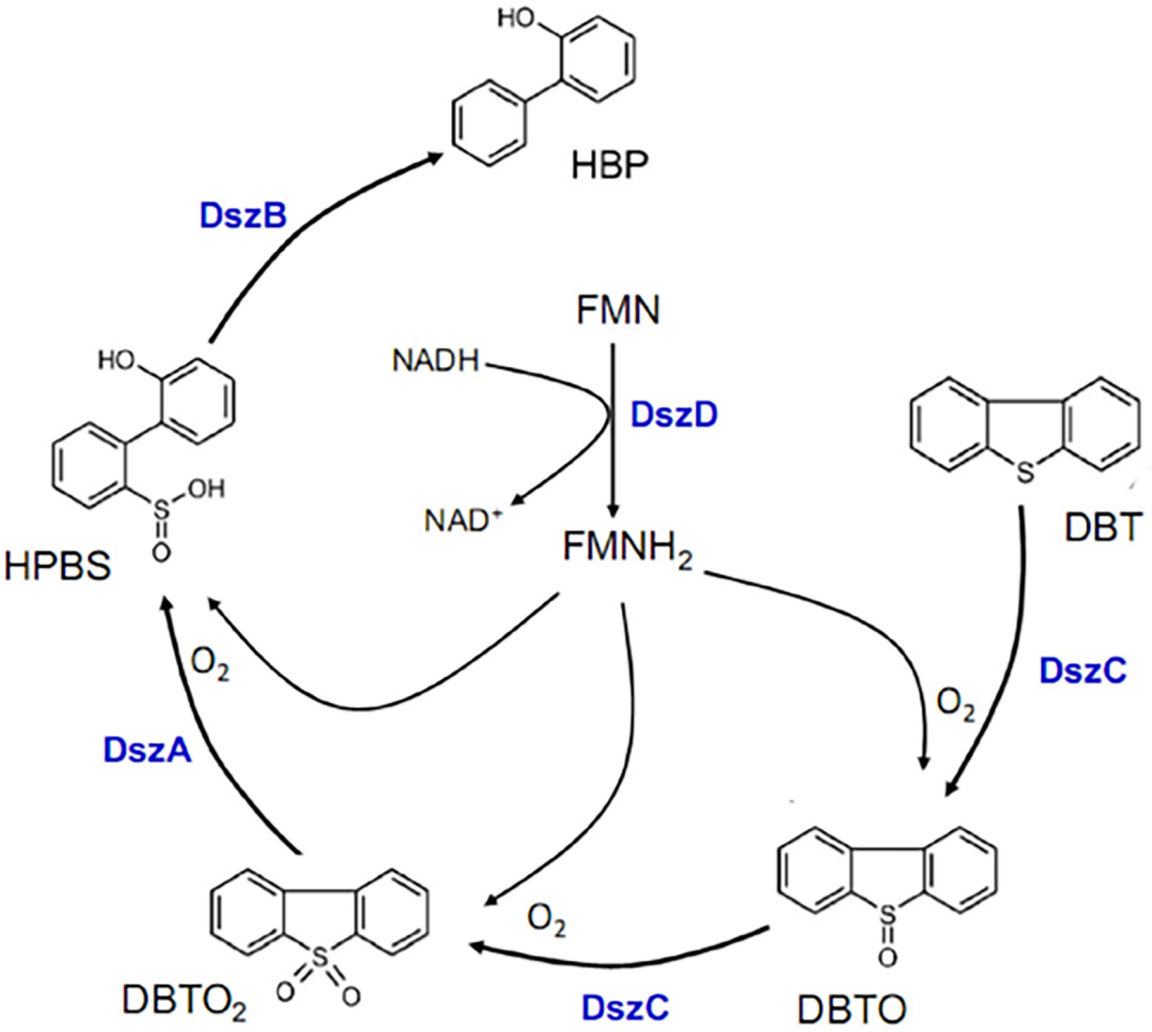
4S-Pathway of biodesulfurization of dibenzothiophene (DBT). Four enzymes (DszA, DzsB, DszC and DszD) are involved in the pathway where the first three steps catalyzed by flavin mononucleotide reduced (FMNH_2_)-dependent monooxygenases, those leading to DBT-sulfoxide (DBTO), DBT-dioxide (DBTO_2_) and hydroxyphenyl benzenesulfinate (HPBS), respectively. The final desulfurization step to 2-hydroxybiphenyl (2-HBP) is catalyzed by desulfinase.

Biodesulfurization of dibenzothiophene and its derived compounds have caught global attention owing to unavailability of economic and eco-friendly techniques for the removal of organic sulfur unlike pyritic sulfur removal. Certain microorganisms have the ability to derive sulfur from complex organic sulfur compounds (like DBT) for their growth and vital activities, and hence can be exploited for organic sulfur removal in fossil fuels with minimal environmental impact [6]. Most of the reported strains oxidize sulfur in DBT by following 4-S pathway with the help of three enzymes, DBT monooxygenase (DszC), DBT sulfone monooxygensae (DszA) and 2-HBP desulfinase (DszB) supported by another enzyme flavin reductase (DszD) as presented in Fig 1 [7–9]. This sulfur specific pathway which does not interrupt the carbon skeleton and proceeds at normal physical conditions is governed by *dsz*ABC operon consequently making it an essential criterion for a potential biodesulfurizing candidate. Various strains like *Rhodococcus erythropolis* IGTS8, *Rhodococcus sp*. Eu-32, *Gordonia alkanivorans* RIPI 90A, *Gordonia alkanivorans* 1B, *Actinomycete sp*., *Gordonia* IITR having *dsz*ABC operon and *dsz*D genes have been reported for biodesulfurization of DBT and its derivatives in last two decades [10–15]. However, not much attention has been paid to explore the differential biodesulfurization of DBT by bacterial strains with reference to the *dsz*ABC operon. Against this background, the present investigation has been designed to explore novel strains of Microbial Type Culture Collection (MTCC) containing *dsz* genes for their ability to desulfurize DBT and compare their end metabolites. This comparative study explains how the different metabolites of DBT are directly linked to the organization of *dsz* genes.

It is also established that DBT enters into the bacterial cell and gets transformed into 2-HBP by the action of *Dsz* enzymes and finally released into the medium [16]. Therefore, in a liquid system like petroleum or oil, DBT is more easily available for the bacteria than that of coal, where DBT is covalently bound to the coal matrix [17]. Keeping these observations in mind we hypothesized that differential availability of free DBT in different sources is contributing for differential behavior of 4S-pathway which is central for the removal of organic sulfur from coal as well as oil without compromising the calorific value. To test this hypothesis, different MTCC strains were explored for desulfurization of DBT.

## Materials and methods

### Materials

Dibenzothiophene (DBT), 2-Hydroxybiphenyl (2-HBP) and glycerol were obtained from Sigma. HPLC grade of ethyl acetate was obtained from SRL. Beef extract, peptone and sodium chloride were obtained from Merck (Darmstadt, Germany). Yeast extract, ammonium chloride and agar were purchased from Hi-media. All Other chemicals were obtained from SRL.

### *In-silico* approach for screening of organisms

Since *Rhodococcus erythropolis IGTS8* (Accession: L37363) has been extensively studied for biodesulfurization of DBT, the nucleotide sequences of *R*. *erythropolis IGTS8* were retrieved from NCBI and explored to find novel biodesulfurizing strains using BLAST. A short sequence stretch 5’ aagtactaccaacacatcgcccgtactctggagcgcggcaagttcgatctgttgtttctg 3’ starting from 901 to 960 of the whole sequence which is part of the *dszABC* operon was used as a query sequence for nucleotide blast program. As an outcome, a list of organisms was obtained from the NCBI database which had *dsz* genes (Table 1). Database hits that had a higher score, expect value, identity (more than 85%) and query coverage (more than 65%) were taken as putative homolog.

**Table 1:** Possible bacterial strains containing *dszABC* operon.

Sl. No.	Accession Number	Organism with *dszABC* operon
1	EF570781.1	Synthetic construct
2	DQ444325.1	*Rhodococcus sp*. *DS-3*
3	AY789136.1	*Rhodococcus sp*. *SDUZAWQ*
4	AY714059.1	*Nocardia globerula*
5	AY714058.1	*Rhodococcus erythropolis*
6	AY714057.1	*Gordonia alkanivorans*
7	AY294402.1	*Rhodococcus erythropolis*
8	AY278323.1	*Rhodococcus sp*. *XP*
9	DQ062154.1	*Acidovorax delafieldii*
10	DQ062161.1	*Brevibacillus brevis*
11	AY960127.1	*Agrobacterium tumefaciens*
12	AJ514948.1	*Rhodococcus sp*. *FMF*
13	U08850.1	*Rhodococcus sp*.
14	L37363.1	*Rhodococcus sp*.
15	EU364831.1	*Gordonia alkanivorans*
16	AJ973326.1	*Gordonia sp*. *RIPI*
17	AB076745.1	*Bacillus subtilis*
18	AY396519.1	*Gordonia sp*. *CYKS2*

### Selection of MTCC cultures

In continuation to the *in-silico* screening, MTCC strains *Rhodococcus rhodochrous* (3552), *Rhodococcus erythropolis* (3951), *Arthrobacter sulfureus* (3332), *Brevibacillus brevis* (6640), *Brevibacillus laterosporous* (2298), *Gordonia alkanivorans* (4014), *Gordonia rubropertincta* (289), *Bacillus subtilis* (1427), *Bacillus subtilis* (2422), and *Acidovorax facilis* (1198) belonging to seven different genera were selected based on their biochemical features (S1 Table) and procured from MTCC, Chandigarh, India). The selection of bacterial strains was based on the criteria that, the MTCC strains were of same genus and species as that of organisms obtained by the *in-silico* approach. Moreover, species that showed the presence of known characteristics like degradation of xenobiotics, phenolics, etc. were preferred. Source of isolation such as from coal mines or oil spill contaminated soil was also given emphasis while selecting novel MTCC strains.

### Enrichment of MTCC culture with DBT

After the cultures were revived in the media prescribed by MTCC, they were grown in minimal salt medium (MSM) containing 2.44 g of KH_2_PO_4_, 14.04 g of Na_2_HPO_4_, 2.0 g of NH_4_Cl, 0.2 g of MgCl_2_, 2.5 mg of MnCl_2_, 1.0 mg of FeCl_3_, 1 mg of CaCl_2,_ and 4 ml of glycerol in 1L of double distilled water (DDW) with pH 7.6 [18]. To check whether the organisms were able to uptake sulfur from DBT, we took sulfur-free MSM medium with different concentration of DBT 0.1, 0.25 and 0.5 mM. Desulfurizing strains growing with a higher concentration of DBT (0.5 mM) were selected for further study. These cultures were incubated for ten days and subjected to screening of *dsz* genes and desulfurizing assay.

### Screening of *dszA*, *dszB*, *dszC* and *dszD*

Total DNA was isolated using a rapid protocol described in literature [19]. In brief, 100 μl of fresh culture was taken in duplicate and centrifuged at 6000xg for 3 min at 4°C. Supernatant containing media were carefully taken out using a pipette. Then 100 μl of autoclaved MilliQ water was added to the pellet, cells were resuspended, thoroughly mixed and incubated at 97°C for 10 min. Cell debris was removed by centrifuging at 10000xg for10 min, at 4°C. The supernatant containing DNA was taken for polymerase chain reaction (PCR). Presence of desulfurizing genes viz. *dszA*, *dszB*, *dszC*, and *dszD* in different cultures were studied by PCR using a BioRad thermal cycler. Gene specific primer sequences from earlier reports were custom synthesized by Integrated DNA Technology, USA. The optimum annealing temperature was examined by taking five different temperatures for the amplification of different PCR product. Information on primers and PCR condition for different genes is given in S2 Table.

The PCR mixture of 25 μl included 0.5 μl total DNA as template, 2.5 μl 10x buffer, 0.2 mM dNTPs, 1.5 mM MgCl_2_, 1U *Taq* DNA polymerase (Fermentas), and 25 pmol of each primer. The mixture was then subjected to PCR with a cycle parameter; 95°C for 3 min, 35 cycles of 94°C for 30s, annealing for 30s, 72°C for 1.5 min and final extension at 72°C for 5 min. The PCR products were electrophoresed on ethidium bromide stained 1.0% agarose gel and images were documented in Cell Biosciences Gel Documentation system. Selected PCR products were sequenced by direct genomic sequencing method for further studies.

### Desulfurization of DBT and analysis of metabolites

Peaks of intermediate metabolites and 2-HBP of the desulfurization process of DBT were observed through HPLC. For the HPLC analysis, ten days old cultures were taken and metabolites were extracted with double the volume of ethyl acetate. The mixture was vigorously shaken in a separating funnel for extraction. After phase separation, lower phase containing the biomass was discarded. The top layer containing ethyl acetate with the organic compound was concentrated to about 0.5 ml to 1 ml by rotary evaporator. Both DBT and 2-HBP were quantified using HPLC (Waters) equipped with NOVA PAK C18 column at 260 nm. The mobile phase was 60% acetonitrile-water, and the flow rate was 1 ml min^-1^. A blank sample without bacterial extract was also analyzed through HPLC. Each sample was run for 30 minutes.

### Phylogenetic analysis of *dsz* operon

Protein sequences for *dsz* operon genes from *Rhodococcus erythropolis IGTS8* were queried against NCBI nt-nr database for bacteria (taxid:2) using tblastn utility. A match was considered if match covers 60% of query sequence with E-value 1E-05 or less. Genes were considered in operonic structure if the gap between genes was 200 nt or less. The evolutionary history was inferred by using the Maximum Likelihood method based on the JTT matrix-based model created from MUSCLE facilitated multiple sequence alignment. The tree with the highest log likelihood (-3696.8650) is shown. The analysis involved 44 amino acid sequences. All positions containing gaps and missing data were eliminated. There were a total of 266 positions in the final dataset. Evolutionary analyses were conducted in MEGA7.

## Results and discussion

### MTCC Cultures and enrichment with DBT

Though a diverse group of bacterial strains have been explored for the desulfurization studies still there is a need to identify more efficient organisms with enhanced desulfurizing ability [7]. In this context we tried to identify the potential microbes by using bioinformatics tools as an easy and quick approach. Initial strain selection was based on BLAST searches using a query sequence consisting of a short fragment of the *dsz*ABC operon against the NCBI database. Selection of strains from the MTCC was then based on similarity of genera names and previous phenotypic information that further indicated competency of strains with greater desulfurizing ability. Based on the *in-silico* criteria and special biochemical characteristics shown by the MTCC strains, around ten microbes from MTCC-IMTECH, Chandigarh (MTCC No. 3552, 3951, 3332, 289, 1198, 6640, 2298, 1427, 2422, and 4014) were used for the present investigation. For enriched culture, the media were supplemented with glycerol and various concentrations i.e. 0.1, 0.25, and 0.5 mM of DBT. Since DBT was the only sulfur source, organisms that can utilize DBT for their growth would capable of desulfurization. As desired, MTCC 3552, 3951, 3332, and 289 strains were able to grow in 0.5 mM of DBT. DBT concentration reported for biodesulfurization was 0.1 mM [20] 0.2 mM [18], 0.5 mM [21], 0.8 mM [22] and 3 mM [23], hence the above strains growing in 0.5 mM DBT were used further for biodesulfurization study.

### Amplification and screening of *dsz* genes

**Fig 2:**
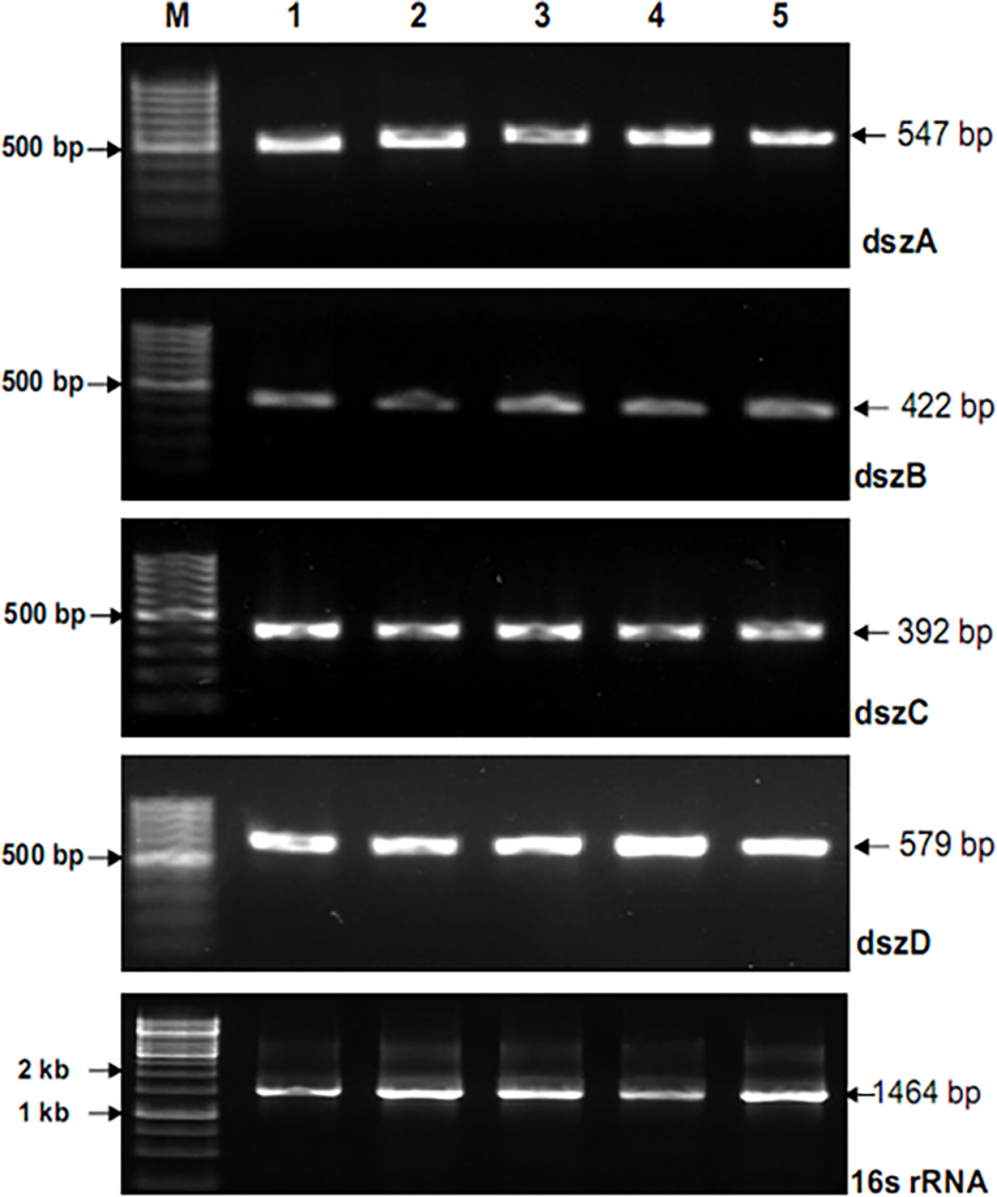
Standardization of PCR product of *dsz* genes and 16s rRNA with different annealing temperatures. The PCR products with different annealing temperature were electrophoresed in agaraose gel against 100 bp DNA ladder.

**Fig 3:**
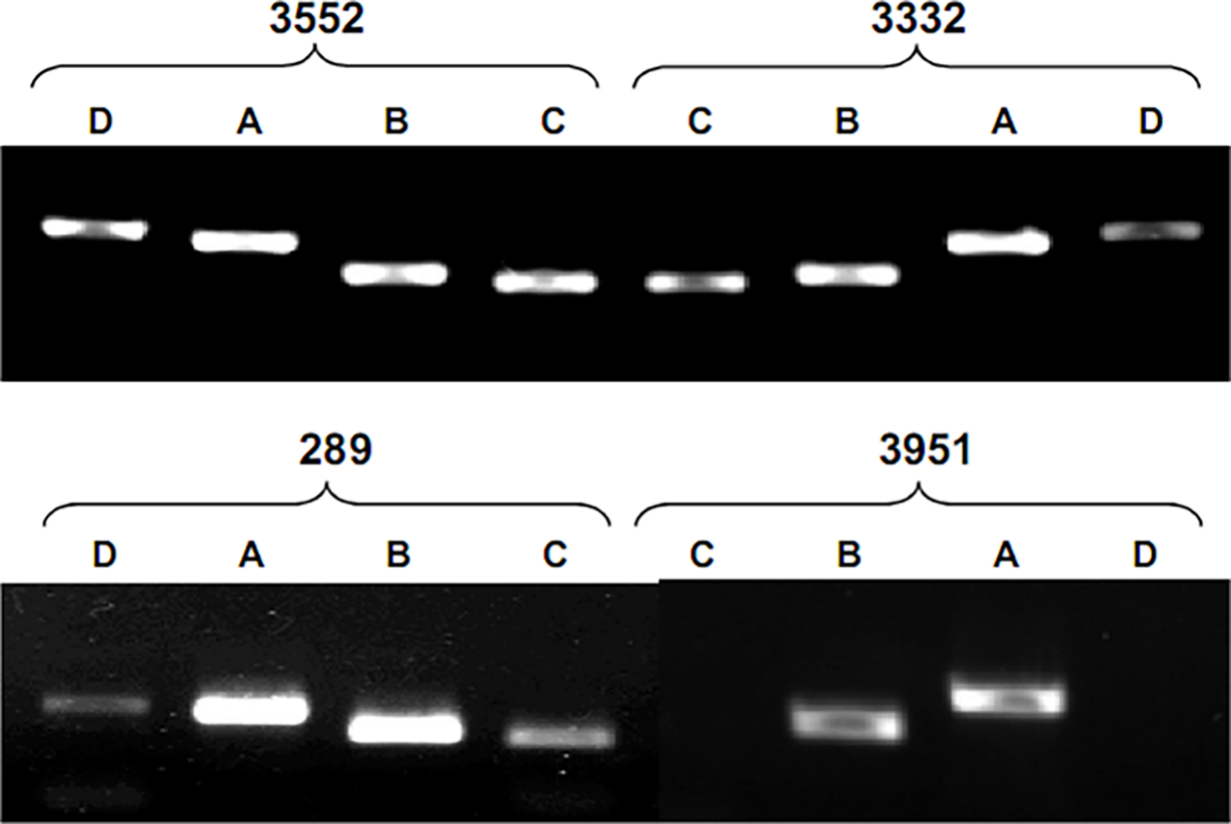
Screening of *dsz* genes in different MTCC strains *Rhodococcus rhodochrous* (3552), *Artrobacter sulfureus* (3332), *Gordonia rubropertincta* (289) and *Rhodococcus erythropolis* (3951) enriched with DBT. The different lanes are showing the amplified products of different genes *dszA*, *dszB*, *dszC* and *dszD*.

Amplification parameters of *dsz* genes were standardized and amplicons were as per expectation with no secondary products. Since the presence of *dsz* operon is a prerequisite for the BDS process in the 4S-pathway, potential MTCC cultures was screened for the presence of *dsz* genes. For this purpose, bacterial DNA of MTCC 3552, 3951, 3332, and 289 were used, and *dsz* genes were screened using gene specific primers (Fig 2). Amplification of 16S rRNA was also carried out as an internal standard present in all the bacteria. Interestingly, all the bands were visible in MTCC strains 3552 and 3332 suggesting both the organisms are capable of producing *Dsz* enzymes which proves highly conserved nature of *dsz* genes [21, 24]. In both the cases, the band intensity of amplified product of *dszD* is low as compared to *dszA*, *dszB*, and *dszC* (Fig 3). Nevertheless, the intensity of *dszD* in 3332 is lower as compared to 3552. Similar pattern was also observed with sample MTCC 289, but the intensity of *dszC* and *dszD* was much lower as compared to *dszA and dszB* (Fig 3). Interestingly with MTCC 3951 the PCR products of *dszA and dszB* were visible without *dszC and dszD*. Lack of the conserved sequences at the beginning of the *dszC* gene could be the explanation of the unsuccessful amplification of *dszC* [12]. From the *dsz* screening experiment it was observed that *dszC* and *dszD* are less abundant than *dszA* and *dszB* in the used strains. The partial sequences of *dszA* and *dszB* genes of strain 3552 and 3332 were aligned against the homologous sequences of reported strains using BIOEDIT graphic interface (S1 Fig). The strain MTCC 3552 shows 99% sequence identities and zero E value with strain *Rhodococcus sp*. DS-3 (genebank id DQ444325.1) and *Norcadia globerula* (genebank id AY714059.1) which are reported for biodesulfurization of diesel oil [24, 25], while MTCC 3332 shows 95% sequence similarity with the above strains (S1 Fig).

### Desulfurization assay by HPLC

**Fig 4:**
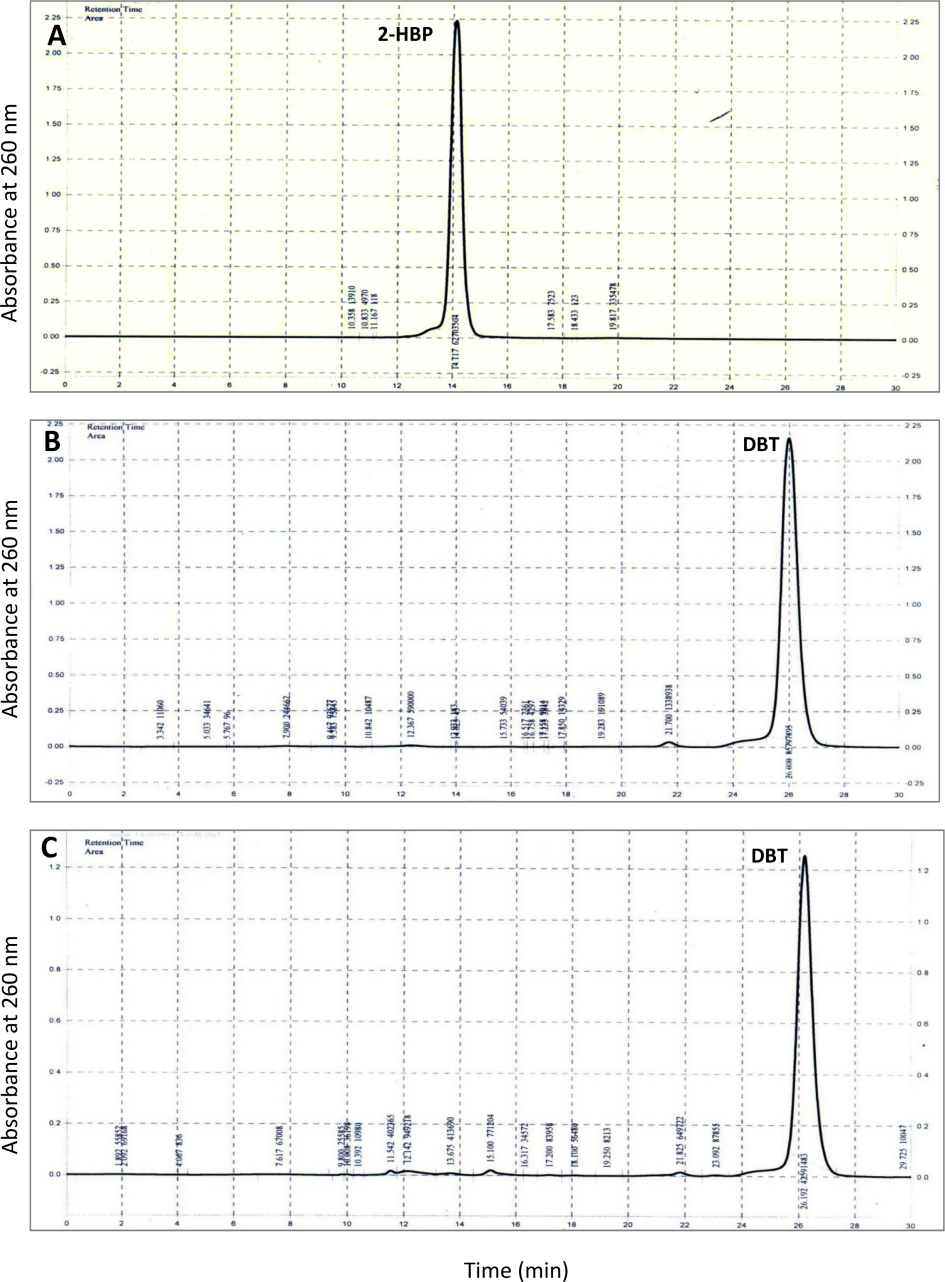
HPLC graph showing the retention time of (a) DBT and (b) 2-HBP. (c) The control DBT without microorganism also shows same retention time with respect to the standard DBT without any degradation.

**Fig 5:**
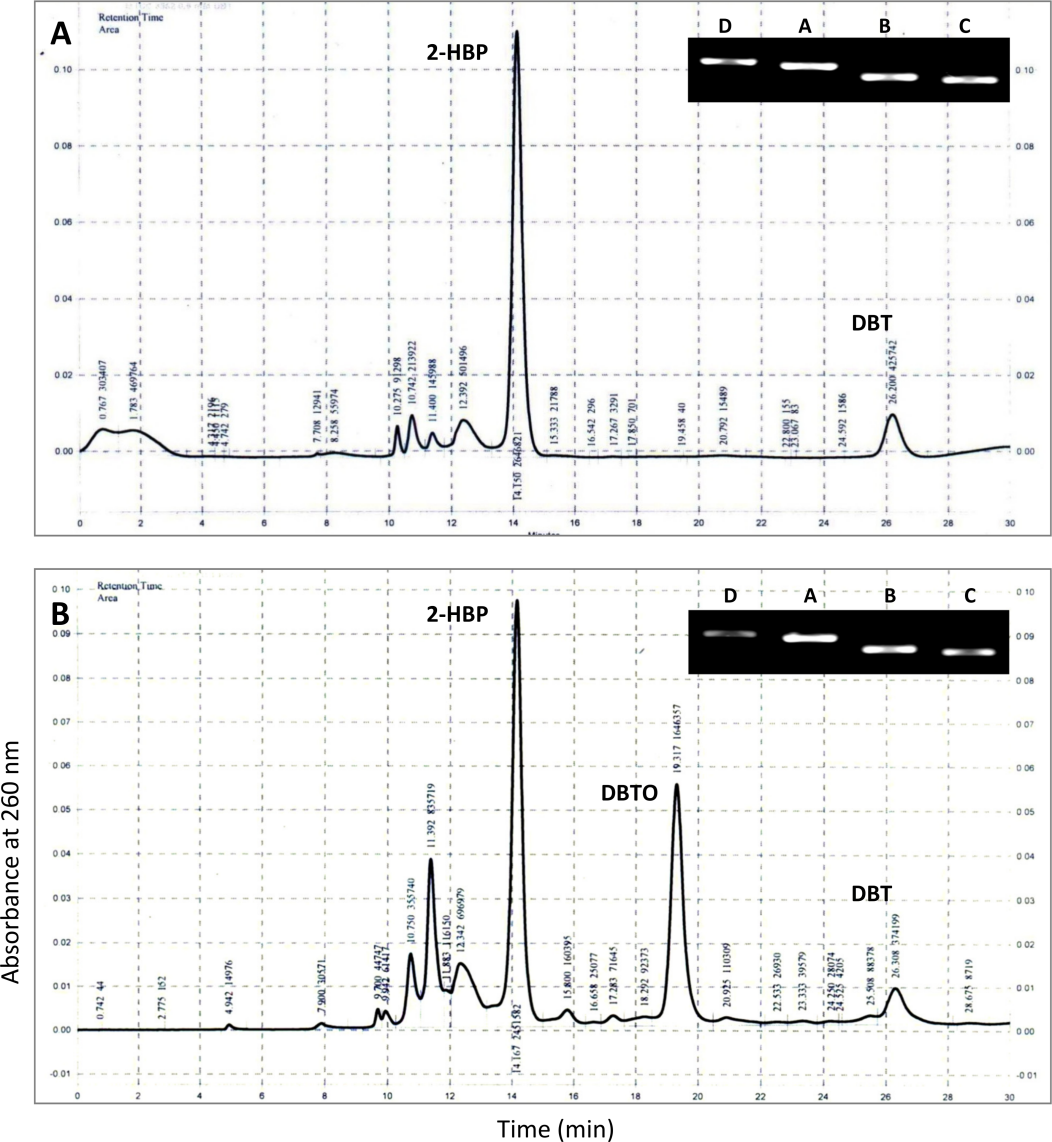
Chromatogram showing the DBT desulfurization after 10 days of growth with different MTCC strains (a) *Rhodococcus rhodochrous* (3552), (b) *Artrobacter sulfureus* (3332), (c) *Gordonia rubropertincta* (289) and (d) *Rhodococcus erythropolis* (3951).

Since *dszABC* operon is central to the biodesulfurization process, it is imperative to understand how *dsz* genes operate in different organisms. With this background, four different MTCC strains (3552, 3951, 3332, and 289) containing *dsz* genes were explored for BDS of DBT and the end products were analyzed by HPLC. Both DBT and 2-HBP standards were prepared in ethyl acetate and injected for the generation of chromatogram. It was found that the retention time of 2-HBP and DBT is 14.125 min and 26.008 min respectively, at 260 nm (Fig 4A and 4B). A chromatogram of DBT (without any degradation) was obtained for the control sample which contained MSM supplemented with 0.5 mM DBT without any organism (Fig 4C). Chromatogram of respective samples were compared and found that the desulfurization of DBT was 98.9%, 99.26%, 99.4%, and 99.63% for strains *Rhodococcus rhodochrous* (3552), *Artrobacter sulfureus* (3332), *Gordonia rubropertincta* (289) and *Rhodococcus erythropolis* (3951) respectively (Fig 5). The most conspicuous observation was that except MTCC 289 all the other organisms showed the presence of 2-HBP, the end product of 4S-pathway. Moreover, a strong peak is appearing with retention time 1.742 min after biodesulfurization of DBT in case of MTCC 3951.

Reported experiments with several organisms along with the organisms used in the present studies with different rates of desulfurization of DBT are listed in Table 2. The strains MTCC 289, 3951, 3332 and 3552 are examined for the first time for BDS studies. Though strains like *Gordonia alkanivorans* with 90% desulfurization of DBT [26], and 70% desulfurization rate [27] has been reported previously but *Gordonia rubropertincta* (MTCC 289) of the present study is having a higher rate 99.4% of desulfurization. Similarly, the organism *Rhodococcus erythropolis* (MTCC 3951) in present study unlike other reported *Rhodococcus erythropolis* strains [21, 28, 29] shows variation in the regular 4S-pathway with a different end product other than 2-HBPS. The organism *Arthrobacter sulfureus* (MTCC 3332) however is showing similar pattern with that of 4-S pathway but with a higher desulfurization rate that is 99.3% than previously investigated *Arthrobacter sulfureus* species with 50% [30] and 83% [31] removal of sulfur. However, strain *Rhodococcus rhodochrous* (MTCC 3552) is showing similar biodesulfurization profile as reported in *Rhodococcus erythropolis IGTS8* [4, 32].

**Table 2:** List of organisms comparing their desulfurizing efficiency in percentage removal of sulfur from literature with reference to the present study.

Reported Strains	Desulfurizing Efficiency	Days of Incubation	References
*Arthrobacter sulfureus*	50%	15	Labana et al. 2005 [30]
*Lysinobacillus sphaericus*	60%	15	Bahuguna et al. 2011 [33]
*Enterobacter spp*. NISOC-03	64%	10	Papizadeh et al. 2016 [22]
*Microbacterium strain ZD-M2*	70%	5	Li et al. 2010 [25]
*Gordonia alkanivorans* 1B	77%	7	Alves et al. 2008 [11]
*Rhodococcus erythropolis* IGTS8	80%	1	Caro et al. 2007 [28]
*Arthrobacter sp*. *P1-1*	83%	14	Seo et al. 2006 [31]
*Sulfolobus solfataricus* P2	88.5%	10	Gun et al. 2015 [20]
*Gordonia alkanivorans RIPI90A*	90%	10	Mohebali et al. 2008 [26]
*Rhodococcus rhodochrous* (MTCC 3552)	**98.9%**	10	This study
*Arthrobacter sulfureus* (MTCC 3332)	**99.26%**	10	This study
*Gordonia rubropertincta* (MTCC 289)	**99.4%**	10	This study
*Rhodococcus erythropolis (MTCC 3951)*	**99.6%**	10	This study
*Rhodococcus erythropolis* SHT87	100%	3	Davoodi-deheghani 2010 [22]

The desulfurization of DBT into 2-HBP by MTCC strains 3552 and 3332 is in accordance with previous findings such as *Rhodococcus sp*. [2, 18], *Lysinibacillus sphaericus* DMT-7 [33], and *Pseudomonas sp*. [34–36]. HPLC chromatogram for both DBT degradation and 2-HBP production is also alike. In addition to the 2-HBP peak in MTCC 3332 one prominent peak with retention time, 19.317 min was also observed. Nevertheless, this particular peak was very intense with the purified metabolites generated by strain MTCC 289 without any 2-HBP peak. Possibly this is an intermediate metabolite DBTO (Fig 1) accumulated in the bacterial cell. From the HPLC profile of MTCC 3951, we find less 2-HBP but the major product is coming with a retention time 1.742 min. However, it is worthy to mention that with 3951, the degradation of DBT was highest among all.

### Phylogenetic analysis of *dsz* operon

The phylogenetic analysis reveals various candidate bacteria which contain the *dsz* operon and can be used in desulfurization process of petroleum and coal products (S2 Fig). Among the bacterial kingdom, the operon is predominantly observed in the species of *Actinobacteria* phylum. In this study also all the four MTCC strains belong to this phylum. However, the infrequent distribution of the *dsz* operon in *Proteobacteria* and *Firmicutes* phyla advocates for the scattered pattern of conservation of *dsz* genes suggesting horizontal gene transfer as a potential mode of origin of the operon. This observation is well corroborated with the phyolgenetic analysis of 16s rRNA sequences and deduced aminoacid sequences of *dsz* genes of previously reported strain *Rhodococcus sps* [13]. In the current study, the absence or faint bands of *dszC* and *dszD* suggests variation in the conserved sequences in the *dsz* operon structure. Different intermediate and end products warrants for the divergent evolution of *dsz* genes by strains isolated from different sources. To test our hypothesis we further investigated MTCC strains which were able to desulfurize free-DBT for the BDS of Assam coal which has high organic sulfur [5]. In support of our hypothesis, we could not get any significant desulfurization by these strains (Data not shown). This experiment clearly indicates that though organisms are capable of desulfurizing free-DBT, the coal desulfurization is not mimicking the process. Moreover, a comparative analysis of *dsz* genes, regulatory regions and the respective enzymes of different strains can offer insights regarding bacterial desulfurization at molecular level.

## Conclusions

Present study reports for the first time the exploration of MTCC strains for biodesulfurization of DBT. The results showed that strains 3552, 3332, 289, and 3951 containing *dsz* genes are able to desulfurize > 99% DBT. Higher degree of sequence homology of the partial sequences of MTCC 3552 and MTCC 3332 and the HPLC profile of culture extracts well supported with the 4S-pathway reported in strains *Rhodococcus erythropolis* DS3 and *Norcadia globerula* for desulfurization of diesel and other model oil. Hence, they could be potentially explored for BDS of petrochemicals with different physiological parameters for an improved desulfurization of petroleum derivatives and oil with an advantageous green complementary approach towards low sulfur fossil-fuels. Further studies can infer regarding application of these strains for a sustainable process designing to meet the interminably growing energy demands at the same time maintaining the environmental strictures.

## Supporting information

S1 Fig Graphic view of aligned sequences of MTCC 3552 and MTCC 3332 with the sequences of *Rhodococcus erythropolis gb│*DQ444325.1*│* from position 915 to 1554 and *Norcadia globerula gb│*AY714059.1*│*from the position 1624 to 2264 with 99% identity and 98% query coverage and 0E value. (DOCX)

S2 Fig The phylogenetic tree with the highest log likelihood for selection of probable bio-desulfurizing candidates. (DOCX)

S1 Table MTCC strains used for the screening of *dsz* genes. Out of ten organisms *Rhodococcus rhodochrous* (3552), *Artrobacter sulfureus* (3332), *Gordonia rubropertincta* (289) and *Rhodococcus erythropolis* (3951) were explored for the biodesulfurization study. All these strains were thoroughly characterized with following biochemical features and maintained in the MTCC, Chandigarh.
(DOCX)

S2 Table Accession number, nucleotide sequence, binding position, annealing temperature, and amplified product size of PCR. (DOCX)

## References

[1] Alves L., Paixao S.M., Pacheco R., Ferreira A.F., Silva C.M., Biodesulphurization of fossil fuels: energy, emissions and cost analysis, RSC Adv. 5 (2015) 34047–34057.

[2] Mohebali G., Ball A.S., Biodesulfurization of diesel fuels–Past, present and future perspectives. Int. Biodeterior. Biodegradation. 110 (2016) 163–180.

[3] Bordoloi N.K., Bhagowati P., Chaudhuri M.K., Mukherjee A.K., Proteomics and Metabolomics Analyses to Elucidate the Desulfurization Pathway of *Chelatococcus sp*., PLoS One. 11 (2016) e0153547 doi: 10.1371/journal.pone.0153547 27100386 10.1371/journal.pone.0153547 PMC4839641

[4] Mishra S., Panda P.P., Pradhan N., Satpathy D., Subudhi U., Biswal S.K., et al, Effect of native bacteria *Sinomonas flava 1C* and *Acidithiobacillus ferrooxidans* on desulfurization of Meghalaya coal and its combustion properties. Fuel 117 (2014) 415–421.

[5] Mishra S., Pradhan N., Panda S., Akcil A., Biodegradation of dibenzothiophene and its application in the production of clean coal. Fuel Processing Technol. 152 (2016) 325–342.

[6] Elmi F., Etemadifar Z., Emtiazi G.A., Novel metabolite (1,3-benzenediol, 5-hexyl) production by *Exophiala spinifera* strain FM through dibenzothiophene desulfurization, World J. Microbiol. Biotechnol. 31 (2015) 813–821. doi: 10.1007/s11274-015-1835-0 25752234 10.1007/s11274-015-1835-0

[7] Buzanello E.B., Rezende R.P., Sousa F.M.O., de Lima S.M., Loguercio L.L., A novel *Bacillus pumilus*-related strain from tropical landfarm soil is capable of rapid dibenzothiophene degradation and biodesulfurization, BMC Microbiol. 14 (2014) 1–10. 10.1186/s12866-014-0257-8 PMC4197255 25293673

[8] Gray K.A., Pogrebinsky G., Mrachko T., Xi L., Monticello D.J., Squires C.H., Molecular mechanisms of biocatalytic desulfurization of fossil fuels, Nat Biotechnol. 4 (1996) 1705–1709. 10.1038/nbt1296-1705 9634856

[9] Bhasarkar J.B., Dikshit P.K., Moholkar V.S., Ultrasound assisted biodesulfurization of liquid fuel using free and immobilized cells of *Rhodococcus rhodochrous* MTCC 3552: A mechanistic investigation, Bioresour. Technol. 187 (2015) 369–378. doi: 10.1016/j.biortech.2015.03.102 25863901 10.1016/j.biortech.2015.03.102

[10] Piddington C.S., Kovacevich B.R., Rambosek J., Sequence and molecular characterization of a DNA region encoding the dibenzothiophene desulfurization operon of *Rhodococcus sp*. strain IGTS8., Appl. Environ. Microbiol. 61 (1995) 468–475. 7574582 10.1128/aem.61.2.468-475.1995 PMC167304

[11] Alves L., Melo M., Mendonça D., Simões F., Matos J., Tenreiro R., et al, Sequencing, cloning and expression of the *dsz* genes required for dibenzothiophene sulfone desulfurization from *Gordonia alkanivorans* strain 1B, Enzyme Microb. Technol. 40 (2007) 1598–1603.

[12] Shavandi M., Sadeghizadeh M., Khajeh K., Mohebali G., Zomorodipour A., Genomic structure and promoter analysis of the *Dsz* operon for dibenzothiophene biodesulfurization from *Gordonia alkanivorans* RIPI90A, Appl. Gen. Mol. Biotechnol. 87 (2010) 1455–1461. 10.1007/s00253-010-2605-4 20414649

[13] Akhtar N., Ghauri M., Anwar M., Heaphy S., Phylogenetic characterization and novelty of organic sulphur metabolizing genes of *Rhodococcus spp*. (Eu-32), Biotechnol. Lett. 37 (2015) 837–847. doi: 10.1007/s10529-014-1736-6 25491478 10.1007/s10529-014-1736-6

[14] Khedkar S., Shanker R., Isolation and classification of a soil actinomycete capable of sulphur-specific biotransformation of dibenzothiophene, benzothiophene and thianthrene, J. Appl. Microbiol. 118 (2015) 62–74. doi: 10.1111/jam.12665 25319398 10.1111/jam.12665

[15] Karimi E., Jeffryes C., Yazdian F., Sepahi A.A., Hatamian A., Rasekh B., et al, DBT desulfurization by decorating Rhodococcus erythropolis IGTS8 using magnetic Fe3O4 nanoparticles in a bioreactor. Eng. Life Sci. 17 (2017) 528–535. 10.1002/elsc.201600080 PMC6999300 32624797

[16] Abin-Fuentes A., Leung J.C., Mohamed M.E.S, Wang D.I.C., Prather K.L.J., Rate-limiting step analysis of the microbial desulfurization of dibenzothiophene in a model oil system, Biotechnol. Bioeng., 111 (2014) 876–884. doi: 10.1002/bit.25148 24284557 10.1002/bit.25148 PMC3969791

[17] Marinov S.P., Gonsalvesh L., Stefanova M., Yperman J., Carleer R., Reggers G., et al, Combustion behaviour of some biodesulphurized coals assessed by TGA/DTA, Thermochim. Acta. 497 (2010), 46–51.

[18] Yu B., Xu P., Shi Q., Ma C., Deep Desulfurization of Diesel Oil and Crude Oils by a Newly Isolated *Rhodococcus erythropolis* Strain, Appl. Environ. Microbiol. 72 (2006) 54–58. doi: 10.1128/AEM.72.1.54-58.2006 16391024 10.1128/AEM.72.1.54-58.2006 PMC1352184

[19] Ohnishi A., Abe S., Nashirozawa S., Shimada S., Fujimoto N, Suzuki M., Development of a 16S rRNA Gene Primer and PCR-Restriction Fragment Length Polymorphism Method for Rapid Detection of Members of the Genus *Megasphaera* and Species-Level Identification, Appl. Environ. Microbiol. 77 (2011) 5533–5535. doi: 10.1128/AEM.00359-11 21705538 10.1128/AEM.00359-11 PMC3147432

[20] Gün G., Yürüm Y., Doğanay G.D., Revisiting the biodesulfurization capability of hyperthermophilic archaeon *Sulfolobus solfataricus* P2 revealed DBT consumption by the organism in an oil/water two-phase liquid system at high temperatures. Turkish J. Chem. 39 (2015) 255–266.

[21] Akhtar N., Ghauri M.A., Anwar M.A., Akhtar K., Analysis of the dibenzothiophene metabolic pathway in a newly isolated *Rhodococcus spp*., FEMS Microbiol. Lett. 301 (2009) 95–102. doi: 10.1111/j.1574-6968.2009.01797.x 19824901 10.1111/j.1574-6968.2009.01797.x

[22] Papizadeh M., Roayaei M.A., Motamedi H., Growth-phase dependent biodesulfurization of Dibenzothiophene by Enterobacter sp. strain NISOC-03. Pollution, 3 *(*2017) 101–111.

[23] Davoodi-Dehaghani F., Vosoughi M., Ziaee A.A., Biodesulfurization of dibenzothiophene by a newly isolated *Rhodococcus erythropolis* strain, Bioresour. Technol. 101 (2010) 1102–1105. doi: 10.1016/j.biortech.2009.08.058 19819129 10.1016/j.biortech.2009.08.058

[24] Dai Y., Shao R., Qi G., Ding B.B., Enhanced Dibenzothiophene Biodesulfurization by Immobilized Cells of *Brevibacterium lutescens* in n-Octane–Water Biphasic System, Appl. Biochem. Biotechnol. 174 (2014) 2236–2244. doi: 10.1007/s12010-014-1184-8 25173674 10.1007/s12010-014-1184-8

[25] Li Y., Li J., Wang C., Wang P., Growth kinetics and phenol biodegradation of psychrotrophic *Pseudomonas putida* LY1, Bioresour. Technol. 17 (2010) 6740–6744. 10.1016/j.biortech.2010.03.083 20385485

[26] Mohebali G., Ballb A.S., Rasekha B., Kaytasha A., Biodesulfurization potential of a newly isolated bacterium *Gordonia alkanivorans* RIPI90A, Enzyme Microb. Technol. 40 (2008) 578–584.

[27] Alves L., Paixão S.M., Toxicity evaluation of 2-hydroxybiphenyl and other compounds involved in studies of fossil fuels biodesulphurisation, Bioresour. Technol. 102 (2011) 9162–9166. doi: 10.1016/j.biortech.2011.06.070 21767949 10.1016/j.biortech.2011.06.070

[28] Caro A., Boltes K., Letón P., García-Calvo E., Dibenzothiophene biodesulfurization in resting cell conditions by aerobic bacteria, J. Biochem. Eng. 35 (2007) 191–197.

[29] Maass D., de Oliveira D., de Souza A.A.U., Souza S.M.A.G.U., Biodesulfurization of a System Containing Synthetic Fuel Using *Rhodococcus erythropolis* ATCC 4277, Appl. Biochem. Biotechnol. 174 (2014) 2079–2085. doi: 10.1007/s12010-014-1189-3 25163887 10.1007/s12010-014-1189-3

[30] Labana S., Pandey G., Jain R.K., Desulphurization of dibenzothiophene and diesel oils by bacteria, Lett. Appl. Microbiol. 40 (2005) 159–163. doi: 10.1111/j.1472-765X.2004.01648.x 15715638 10.1111/j.1472-765X.2004.01648.x

[31] Seo J.S., Keum Y.S., Cho L.L.K., Li Q.X., Degradation of dibenzothiophene and carbazole by *Arthrobacter sp*. Int. Biodeter. Biodegr. 58 (2006) 1–1.

[32] Kilbane J.J. II, Microbial biocatalyst developments to upgrade fossil fuels, Curr. Opin. Biotechnol. 17 (2006) 305–314. doi: 10.1016/j.copbio.2006.04.005 16678400 10.1016/j.copbio.2006.04.005

[33] Bahuguna A., Lily M.K., Munjal A., Singh, Dangwal K., Desulfurization of dibenzothiophene (DBT) by a novel strain *Lysinibacillus sphaericus* DMT-7 isolated from diesel contaminated soil, J. Env. Sci. 23 (2011) 975–982. 10.1016/s1001-0742(10)60504-9 22066220

[34] Luo M., Xing J., Gou Z., Li S., Liu H., Chen J. Desulfurization of dibenzothiophene by lyophilized cells of *Pseudomonas delafieldii R-8* in the presence of dodecane. Biochem. Eng. J. 13 (2003) 1–6.

[35] Alcon A., Martin A., Santos V., Gomez E., Garcia-Ochoa F. Kinetic model for DBT desulfurization by resting whole cells of *Pseudomonas putida* CECT5279. Biochem. Eng. J. 39 (2008) 486–495.

[36] Martinez I., Santos V.E., Garcia-Ochoa F. Metabolic kinetic model for dibenzothiophene desulfurization through 4S-pathway using intracellular compound concentrations. Biochem. Eng. J. 117 (2017) 89–96.

