# Correction: Differential desulfurization of dibenzothiophene by newly identified MTCC strains: Influence of Operon Array

**DOI:** 10.1371/journal.pone.0196374

**Published:** 2018-04-19

**Authors:** The PLOS ONE Staff

In Fig 5, panels c and d are missing. The publisher apologizes for the error. The authors have provided a corrected version here.

**Fig 5:**
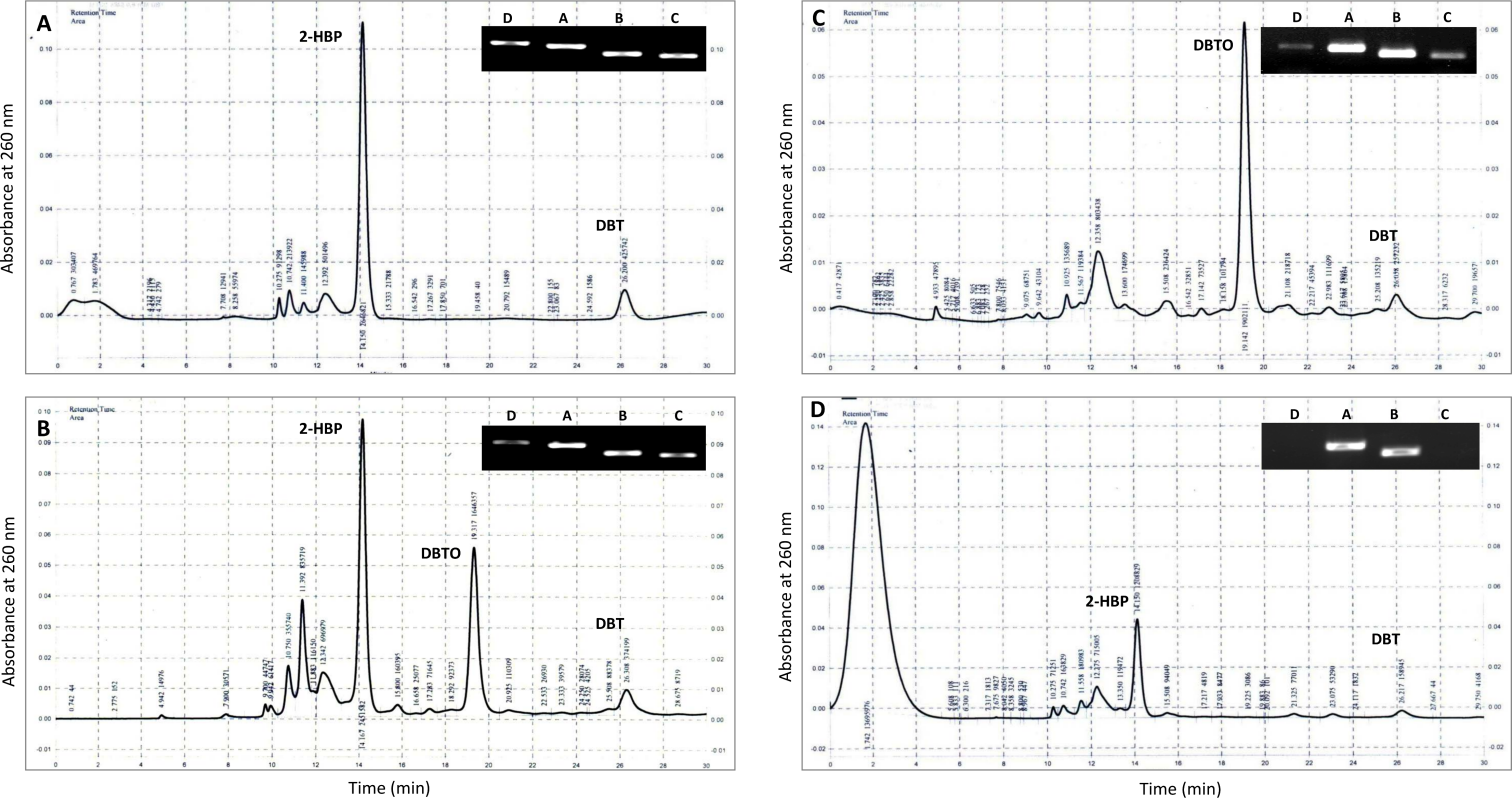
Chromatogram showing the DBT desulfurization after 10 days of growth with different MTCC strains (a) *Rhodococcus rhodochrous* (3552), (b) *Artrobacter sulfureus* (3332), (c) *Gordonia rubropertincta* (289) and (d) *Rhodococcus erythropolis* (3951).
