# Supplementary material for: Differential desulfurization of dibenzothiophene by newly identified MTCC strains: Influence of Operon Array
Source: PLoS One. 2018 Mar 8;13(3):e0192536. doi: 10.1371/journal.pone.0192536 (PMC5843161; doi:10.1371/journal.pone.0192536)

**S1 Fig.** Graphic view of aligned sequences of MTCC 3552 and MTCC 3332 with the sequences of *Rhodococcus erythropolis gb│*DQ444325.1*│* from position 915 to 1554 and *Norcadia globerula gb│*AY714059.1*│*from the position 1624 to 2264 with 99% identity and 98% query coverage and 0E value.


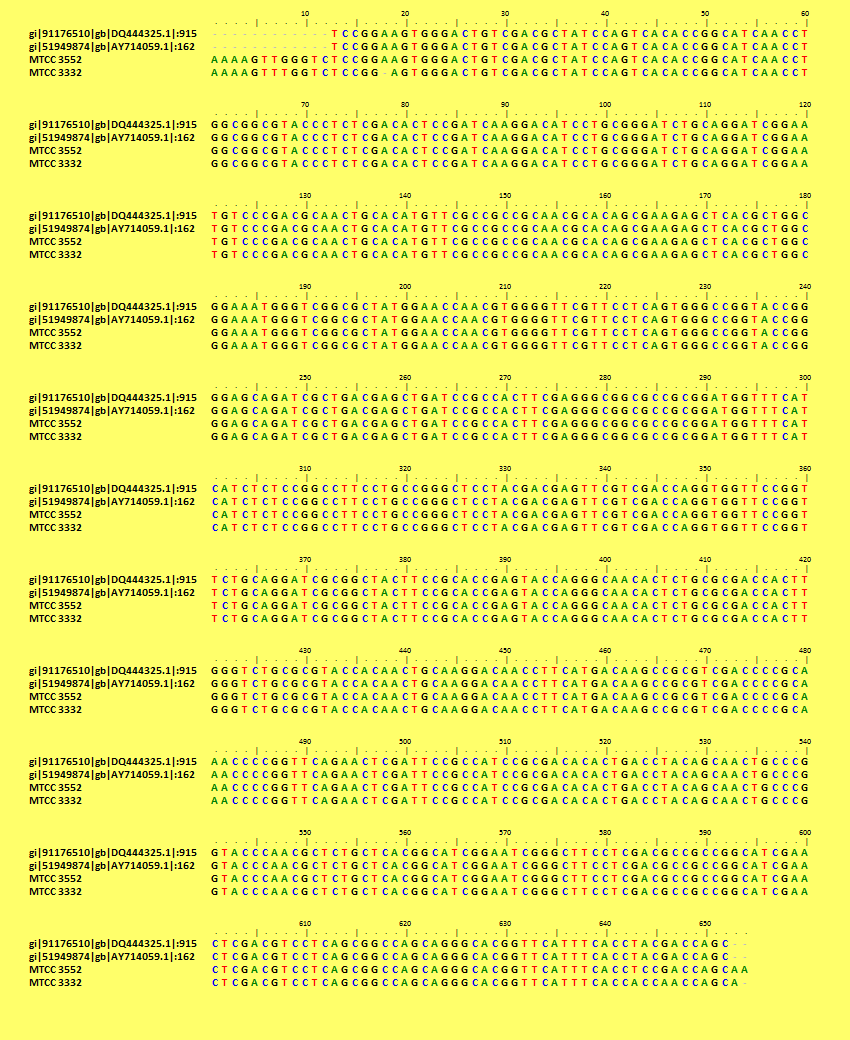


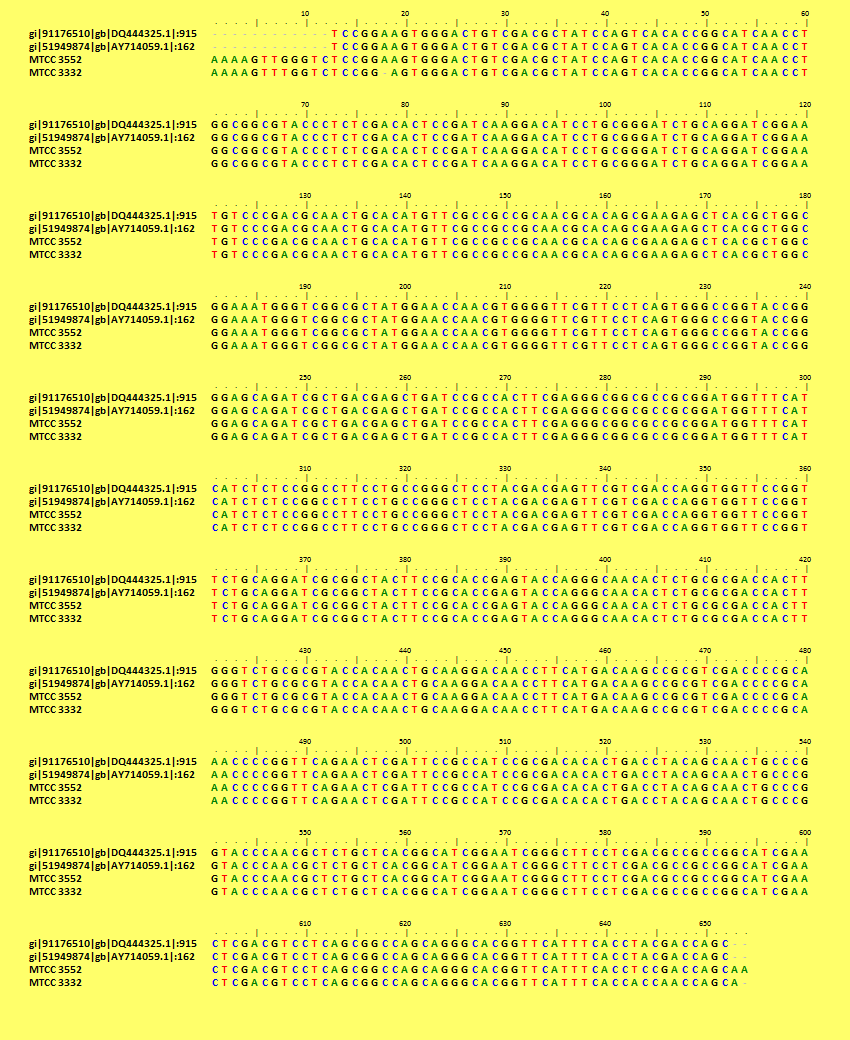

Supplement: S1 Fig — (DOCX) [file pone.0192536.s001.docx]
