# Supplementary material for: Differential desulfurization of dibenzothiophene by newly identified MTCC strains: Influence of Operon Array
Source: PLoS One. 2018 Mar 8;13(3):e0192536. doi: 10.1371/journal.pone.0192536 (PMC5843161; doi:10.1371/journal.pone.0192536)

**S2 Fig.** The phylogenetic tree with the highest log likelihood for selection of probable bio-desulfurizing candidates.


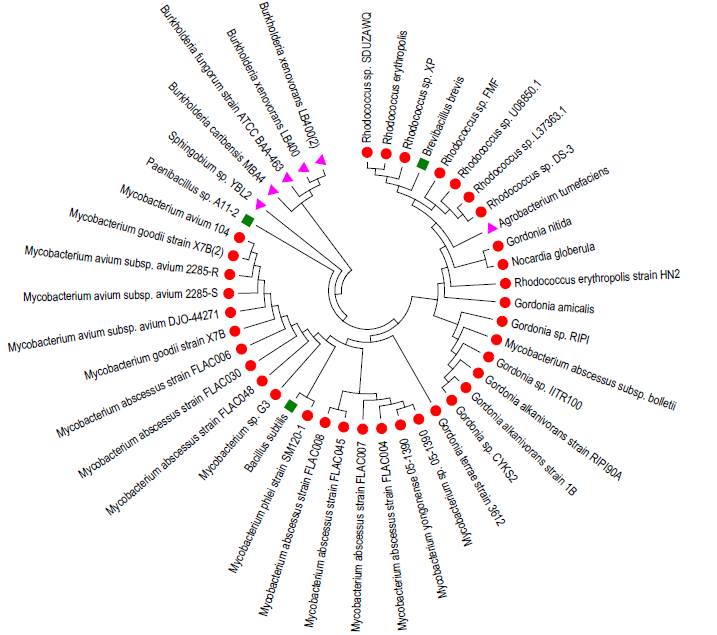

Supplement: S2 Fig — (DOCX) [file pone.0192536.s002.docx]
