# Supplementary material for: Differential desulfurization of dibenzothiophene by newly identified MTCC strains: Influence of Operon Array
Source: PLoS One. 2018 Mar 8;13(3):e0192536. doi: 10.1371/journal.pone.0192536 (PMC5843161; doi:10.1371/journal.pone.0192536)
Supplement: S1 Table — Out of ten organisms Rhodococcus rhodochrous (3552), Artrobacter sulfureus (3332), Gordonia rubropertincta (289) and Rhodococcus erythropolis (3951) were explored for the biodesulfurization study. All these strains were thoroughly characterized with following biochemical features and maintained in the MTCC, Chandigarh. (DOCX) [file pone.0192536.s003.docx]

| **Sl. No.** | **MTCC No.** | **Organism** | **Special Feature** | **Source of Isolation** |
| --- | --- | --- | --- | --- |
| 1 | 3552 | *Rhodococcus rhodochrous* | Cleaves organic C-S bond allowing removal of sulfur from fossil fuel and reduces petroleum viscosity. | Soil |
| 2 | 3951 | *Rhodococcus erythropolis* | Degrades monochlorophenols, dichlorophenol, trichlorophenol, phenol, cresols, catechol, toluene and benzoic acid. | Soil |
| 3 | 3332 | *Arthrobacter sulfureus* | Degradation of phenantherene | Oil contaminated soil swamp |
| 4 | 6640 | *Brevibacillus brevis* | Not specified | Potato rhizosphere |
| 5 | 2298 | *Brevibacillus laterosporous* | Production of biosurfactant | Crude oil tank bottom sludge |
| 6 | 4014 | *Gordonia alkanivorans* | Type strain | Tar and phenol contaminate |
| 7 | 289 | *Gordonia rubropertincta* | Oxidation of hydrocarbons | Propane mineral salt |
| 8 | 1427 | *Bacillus subtilis* | Production of surfactant surfactin | Not specified |
| 9 | 2422 | *Bacillus subtilis* | Production of surfactin | Not specified |
| 10 | 1198 | *Acidovorax facilis* | Chemolithotrophic and growth with hydrogen | Soil |

**S1 Table.** MTCC strains used for the screening of *dsz* genes. Out of ten organisms *Rhodococcus rhodochrous* (3552), *Artrobacter sulfureus* (3332), *Gordonia rubropertincta* (289) and *Rhodococcus erythropolis* (3951) were explored for the biodesulfurization study. All these strains were thoroughly characterized with following biochemical features and maintained in the MTCC, Chandigarh.
