# Supplementary material for: Differential desulfurization of dibenzothiophene by newly identified MTCC strains: Influence of Operon Array
Source: PLoS One. 2018 Mar 8;13(3):e0192536. doi: 10.1371/journal.pone.0192536 (PMC5843161; doi:10.1371/journal.pone.0192536)
Supplement: S2 Table — (DOCX) [file pone.0192536.s004.docx]

**S2 Table**. Accession number, nucleotide sequence, binding position, annealing temperature, and amplified product size of PCR

| **Gene** | **Accession Number** | **Primer Sequence (5́′ – 3′)** | **Binding Position** | **Annealing**  **Temp. ºC** | **PCR Product** |
| --- | --- | --- | --- | --- | --- |
| *dszA* | DQ444325.1 | S – TCG ATC AGT TGT CAG GGG | 371-388 | 49.6 | 547 bp |
|  |  | AS- GGA TGG ACC AGA CTG TTG AG | 917-898 |  |  |
| *dszB* | DQ444325.1 | S- ATC GAA CTC GAC GTC CTC AG | 1497-1516 | 51.4 | 422 bp |
|  |  | AS- GGA ACA TCG ACA CCA GGA CT | 1918-1899 |  |  |
| *dszC* | DQ444325.1 | S- CTG TTC GGA TAC CAC CTC AC | 2743-2762 | 50.4 | 392 bp |
|  |  | AS- ACG TTG TGG AAG TCC GTG | 3134-3117 |  |  |
| *dszD* | DQ44326.1 | S- ATG TCT GAC AAG CCG AAT GCC | 1-21 | 46.8 | 579 bp |
|  |  | AS- TCT AGA CTA TTG ACC TAA CGG | 579-559 |  |  |
| *16S rRNA* | NR_044761 | S- AGAGTTTGATCCTGGCTCAG | 8-27 | 49.6 | 1464 bp |
|  |  | AS- GTTACCTTGTTACGACTTCAC | 1471-1457 |  |  |

**NB:** S and AS denotes Sense and antisense primer of respective genes
